# Neonatal small bowel perforation in ileal atresia in a low resource setting: A case report

**DOI:** 10.1002/ccr3.3323

**Published:** 2020-09-17

**Authors:** Okidi Ronald, Odwar Erick, Aber Lucy Diana, Nahurira Violah, Komakech David

**Affiliations:** ^1^ Department of Surgery St. Mary's Hospital Lacor Gulu Uganda; ^2^ Department of Anesthesia and Critical Care St. Mary's Hospital Lacor Gulu Uganda; ^3^ Department of Pediatrics & Child Health St. Mary's Hospital Lacor Gulu Uganda

**Keywords:** Ileal, neonate, perforation

## Abstract

Jejunoileal atresia is a common cause of neonatal intestinal obstruction with high mortality and morbidity in a low resource setting where surgical care is lacking. Herein, a 4‐day‐old presented with features of acute abdomen and septicemia, managed with ileostomy diversion, and recovered uneventfully.

## Graphical abstract

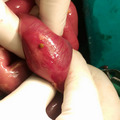

## 1 BACKGROUND

Neonatal surgical care and neonatal intensive care are lacking in Uganda, while jejunoileal atresia and stenosis are still the most common causes of neonatal intestinal obstruction with high mortality and morbidity in a low resource setting. Herein, we presented a 4‐day‐old neonate who presented to the emergency department of St. Mary's Hospital Lacor as a referral from a distant peripheral hospital with features of acute abdomen and septicemia. He was resuscitated and an ileostomy diversion created. He recovered uneventfully from the hospital and discharged. However, he passed on upon readmission six weeks later from severe acute malnutrition. There should be a high index of suspicion on concomitant intestinal perforation in a neonate presenting with intestinal obstruction after delayed surgical care. Surgical approaches such as intestinal diversion should be sought carefully to mitigate serious consequences of anastomotic leak from primary bowel repair.

Neonatal intestinal perforation is one condition with a high mortality rate.^1^ Its etiologies vary with necrotizing enterocolitis being the most common at 43.8%. Among others include Jejunoileal atresia which initially presents with intestinal obstruction, while toddlers and infants may have intussusception causing an intestinal obstruction as well but a rare phenomenon.^2^, ^3^ A systematic review by Sebastian et al, the burden of intestinal atresia, was at 54.9% in the studies of neonatal surgical conditions in Africa with highest mortality >50% in emergency neonatal surgery.^4^ In a retrospective review of babies who had laparotomy for jejunoileal atresia, 69.8% had ileal atresia and of those, 20.9% had type 1 atresia, and meconium peritonitis was in 43.3% of the cases and was considered to be secondary to perforation.^5^ A non‐NEC‐related perforation can be idiopathic, secondary to any underlying pathology or a spontaneous perforation occurring in a normal bowel without any evident cause.^6^ In intestinal obstruction as an underlying pathology mostly presents with abdominal distension and vomiting for 3 to 4 days in 74% of the neonates and commonly affected part of the bowel is the terminal ileum and neonatal gastrointestinal perforation may present with failure to pass meconium, vomiting, abdominal distension, and pneumoperitoneum.^6^, ^7^

A plain radiograph of abdomen continues to be a useful tool for the diagnosis of neonatal intestinal obstruction demonstrating distended bowel loop or double bubble in proximal obstruction secondary to duodenal atresia.^8^, ^9^

The management of neonatal intestinal perforation depends on the etiological factor and varies to include primary repair, resection and anastomoses, ileostomy, colostomy, gastrectomy, and gastroduodenostomy.^10^, ^11^, ^12^ Though, Hiller et al reported neonates with ileal atresia who undergo primary resection and anastomosis had a better outcome than those who had secondary anastomosis, mortality, and major complications are seen in neonates managed with primary anastomoses compared to ileostomy.^13^, ^14^

## 2 CASE DESCRIPTION

A four (4)‐day‐old neonate, born to a 21‐year‐old primiparous mother delivered at term with a birthweight of 2.30 kg, was admitted to the emergency department of St. Mary's Hospital Lacor as a referral from another health unit with failure to pass stool associated with abdominal distension, vomiting of nonbilious content, and irritability since birth. There was no yellowing of eyes or skin, and he was able to breastfeed well and passed urine normally.

### 2.1 Clinical examination

He was ill‐looking, irritable, febrile at 38.3°C, with pink mucosal membranes, and was mildly dehydrated. He was in respiratory distress, respiring at 65 breaths per min with oxygen saturation (SPO_2_) of 89% on room air and a pulse rate of 158 beats per min (bpm). The chest had normal air with normal breath sound and percussion note. The abdomen was grossly distended with visible collateral veins, diffusely tender with guarding. Upon digital rectal examination, there was a normal anus with a normal anal tone, no perineal fistula, and an empty rectum. Noted the absence of blast sign.

### 2.2 Investigation

**Figure 1:**
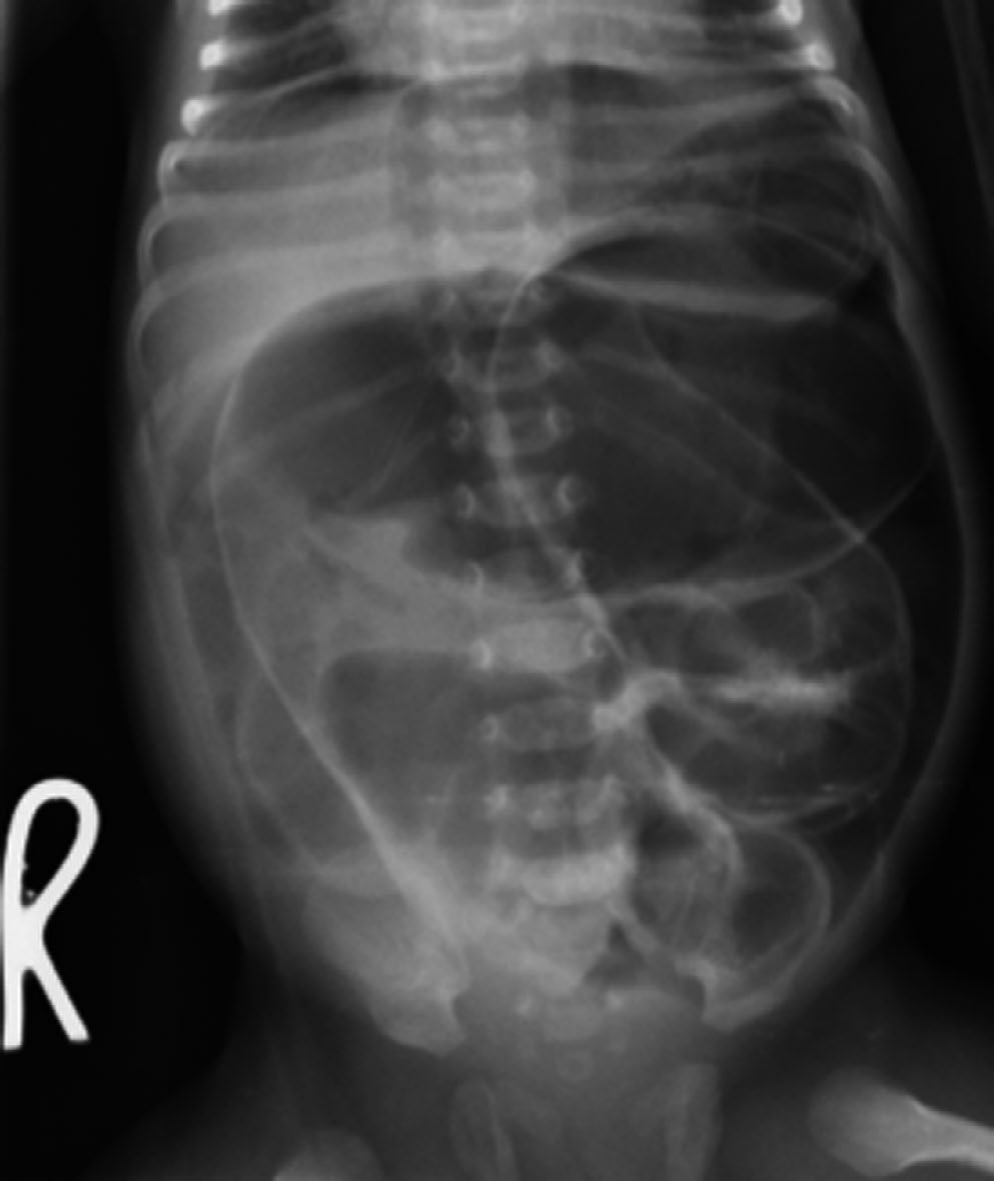
Showing a plain supine abdominal X‐ray of the neonate

Abdominal ultrasonography revealed bowel distension with gas and no peristaltic movement, and a supine abdominal X‐ray revealed grossly distended bowel loops (Figure 1). A full hemogram performed showed leukocytosis and granulocytosis with a normal hemoglobin count (Table 1).

**Table 1:** Showing a panel of laboratory investigations performed

Investigation	Result
Complete blood count	Leukocytes count of 13,380 cells/mL Granulocytes count of 11,010 cells/mL Hemoglobin count of 14.1g/dL Platelets count of 309,000 cells/mL
Blood slide for malaria parasite	No malaria parasites seen

### 2.3 Management

**Figure 2:**
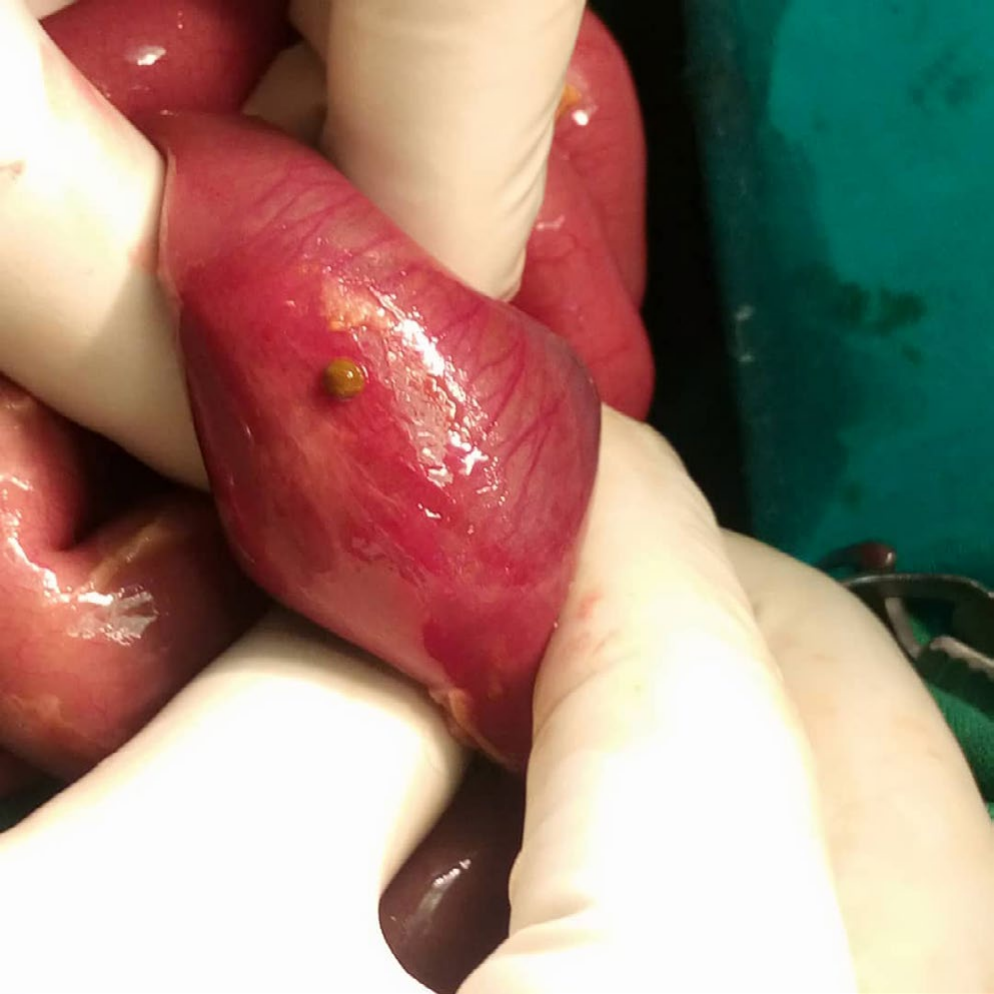
Showing the small bowel perforation site

**Figure 3:**
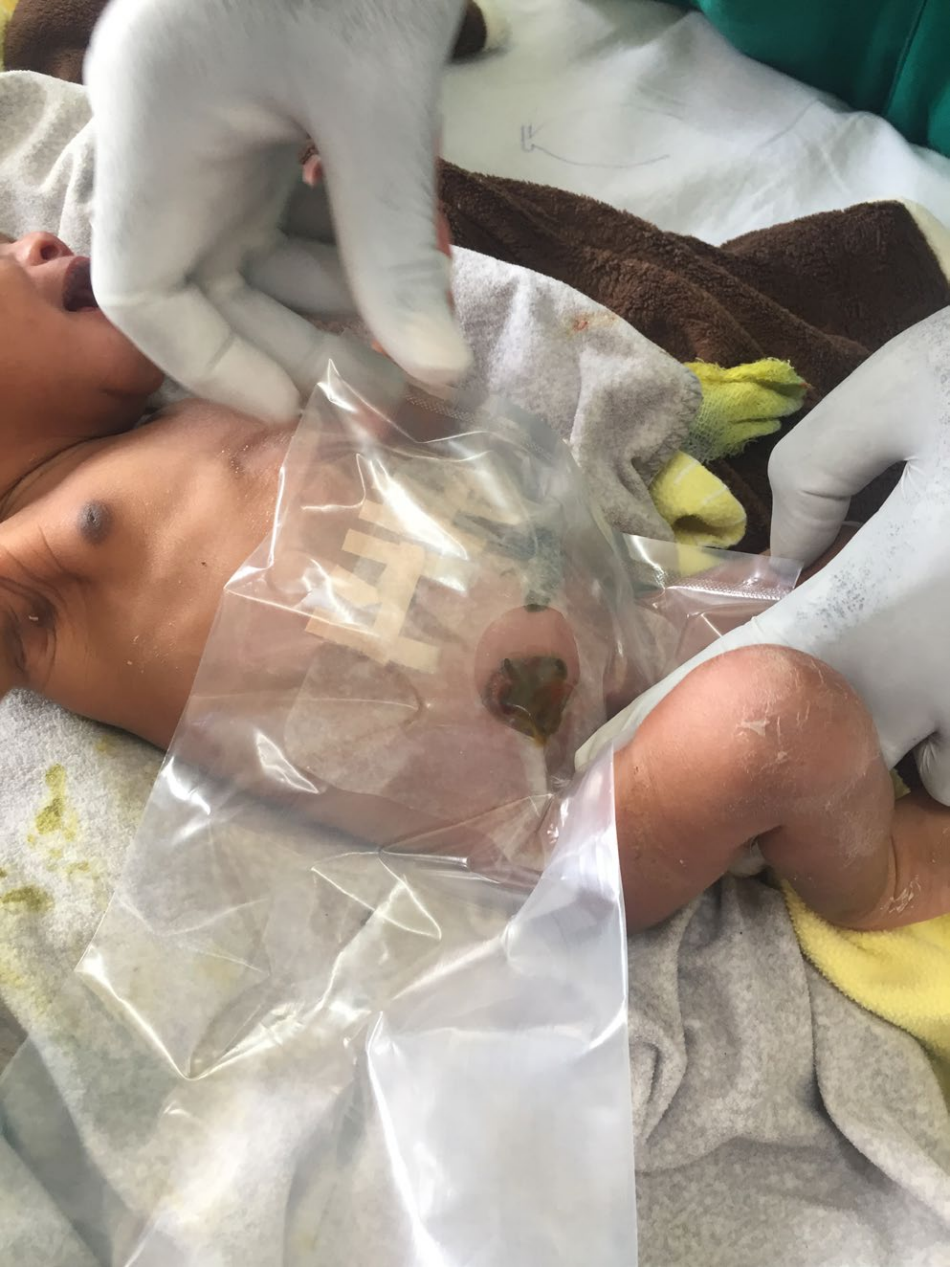
Showing the postoperative ileostomy with a collection bag attached on to the skin

**Figure 4:**
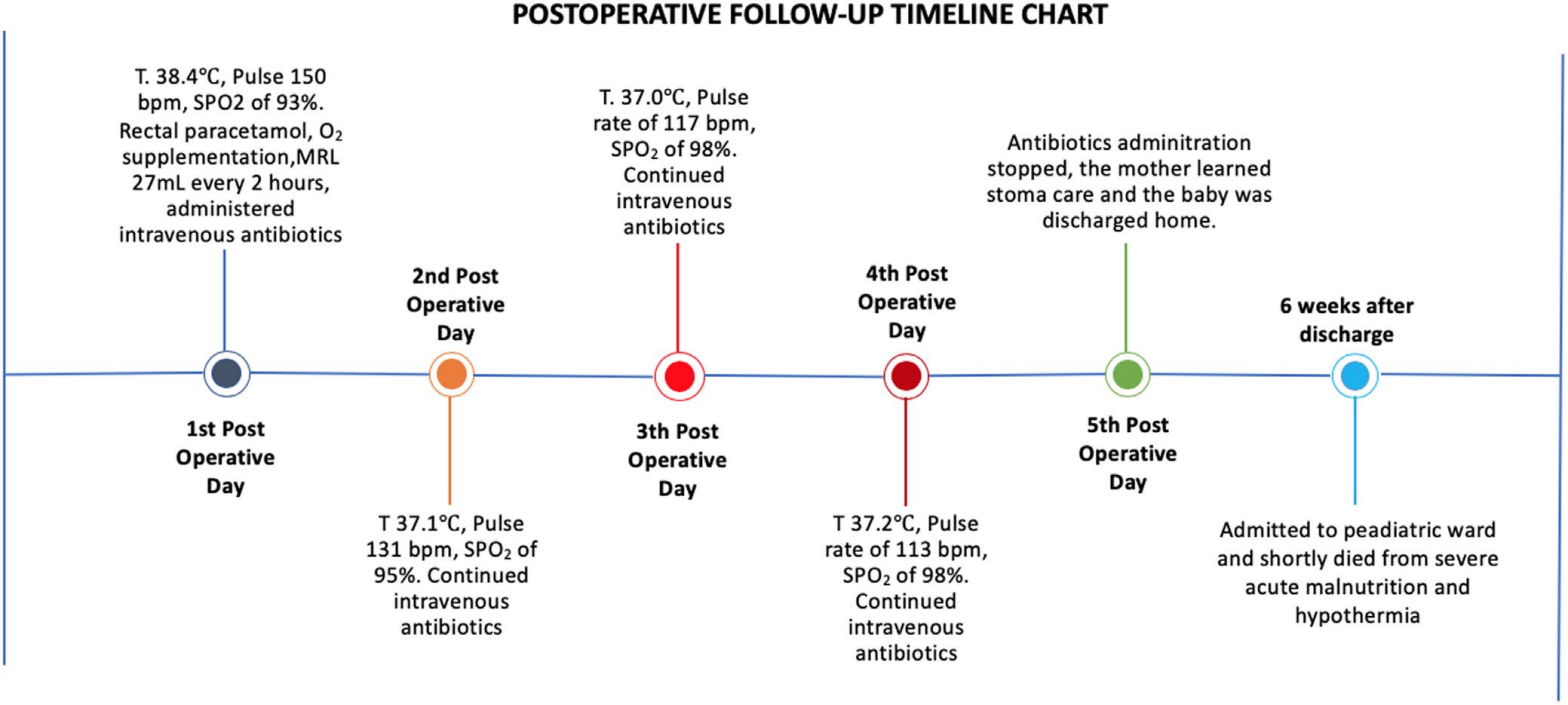
Showing the patient's clinical follow‐up timeline chart

A nasogastric suction tube and a urinary catheter inserted. Intravenous resuscitation was done with modified Ringer Lactate (MRL) and operated. Intraoperative findings of a grossly distended ileum, 15 cm proximal to the ileocecal junction (ICJ) for a length of 16.0 cm, and a perforation 18.0 cm from the ICJ with edematous bowel and intraperitoneal contamination (Figure 2)a collapsed distal bowel with a transluminal septum at the point of obstruction. Resected the 2.0 cm bowel including the septum and created a double‐barrel ileostomy (Figure 3). He was managed in intensive care unit (ICU) postoperatively till discharged with a diagnosis of type I ileal atresia with terminal ileal perforation. Six weeks later, he developed severe acute malnutrition, hypothermia, and dyspnea and passed on in hospital upon readmission (Figure 4).

## 3 DISCUSSION

Neonatal intestinal obstruction is so devastating if access to care is not immediate. This may progress to possible proximal bowel perforation, aspiration, and increased intra‐abdominal pressure compromising breathing as seen in this child who was admitted with an oxygen saturation of 89% room air.^7^ As known, access to proper surgical care is still a huge challenge in developing countries as often are delayed before reaching the point of care. The neonate was referred from a health center, 100‐kilometer distance away already in a complicated state, distressed and hypoxic with probable increased intra‐abdominal pressure secondary to the abdominal distension and bowel edema.^2^, ^3^ The cause of Perforation in these neonates was found to be secondary to a more distal obstruction, and eventual perforation was caused by ileal atresia type I.^5^ The plain abdominal radiographic findings of dilated small bowel greatly supported a diagnosis of intestinal obstruction. However, the possibility of minute perforation could not be excluded since he presented with additional features of peritonitis.^8^, ^9^

The surgical management for ileal atresia reported by Hiller et al is in favor of primary resection and anastomosis than secondary anastomosis.^14^ This neonate was managed by constructing a double‐barrel ileostomy owing to the fact there was gross peritoneal contamination by meconium and bowel edema mitigating the risk of the anastomotic leak.^15^ Consequently, the neonate deteriorated at home following poor, inadequate breastfeeding by the mother and eventually died in the hospital upon readmission from severe acute malnutrition and hypothermia six weeks later.

## 4 CONCLUSION

A high index of suspicion on concomitant intestinal perforation in a neonate presenting with intestinal obstruction after delayed access to surgical care while approaches such as intestinal diversion should be sought carefully to mitigate serious consequences of anastomotic leak from primary bowel repair.

## CONFLICT OF INTEREST

None declared.

## AUTHOR CONTRIBUTIONS

Okidi Ronald: wrote the background, discussion of the case, and preparation of the manuscript; Odwar Erick: participated in the case discussion; Komakech David: added background and summarized the case description, Aber Lucy Diana: provided photographs of the baby and obtained informed consent for publication of the case; and Nahurira violah: was involved in the writing of the abstract. All the authors participated in the management of this case.

## ETHICAL APPROVAL

This case report was approved by the Lacor hospital institutional research and ethics committee (LHIREC).

## INFORMED CONSENT

The mother gave written informed consent for the publication of her baby's clinical information including the photographs.
